# The effect of shrubs admixture in pine forest stands on soil bacterial and fungal communities and accumulation of polycyclic aromatic hydrocarbons

**DOI:** 10.1038/s41598-023-43925-x

**Published:** 2023-10-02

**Authors:** Jarosław Lasota, Rafał Ważny, Marzena Kaźmierczak, Ewa Błońska

**Affiliations:** 1 Department of Ecology and Silviculture, Faculty of Forestry, University of Agriculture in Krakow, 29 Listopada 46 Str., 31-425 Kraków, Poland; 2 Małopolska Centre of Biotechnology, Jagiellonian University in Kraków, Gronostajowa 7a, 30-387 Kraków, Poland

**Keywords:** Biochemistry, Ecology, Biogeochemistry

## Abstract

Polycyclic aromatic hydrocarbons (PAHs) are a group of persistent toxic pollutants. The species composition of the stand is important in shaping the quality of soil organic matter and, consequently, the PAH content. The main purpose of the research was to determine the role of shrubs in shaping PAH accumulation in forest soils. The study covered the soils of the pine stands of the Rybnik Forest District, which experiences some of the highest deposition of industrial emissions in Europe. Pine stands with admixture of shrubs (alder buckthorn *Frangula alnus* and European hazelnut *Coryllus avellana*) growing in the same soil conditions were selected for the study. Samples for analyses were collected from the organic horizon (O) (from a depth of 0–7 cm) and humus mineral horizon (A) (from a depth of 7–15 cm). The organic C and total N concentrations, pH, alkaline cation content, soil enzyme activity and PAH content were determined. Additionally, the taxonomic composition of soil bacterial and fungal communities was determined. The highest activity of enzymes was noted in soils under influence of shrubs. The enzymatic activity was positively correlated with the content of total N, organic C, pH H_2_O and KCl and negatively with the C/N ratio. The highest PAH content was recorded in the soils of pine stands without the admixture of shrubs. Our research indicates the importance of shrubs in shaping the properties of surface horizons of forest soil and, consequently on the accumulation of PAHs. Shrubs stimulate biochemical activity of soils which results in lower PAHs accumulation by providing more easily decomposable organic matter.

## Introduction

In central Europe, pure coniferous stands are viewed negatively today, whereas mixed stands are recommended^1,2^. Pretzsch et al.^3^ observed positive and multiplicative mixing effects on structural heterogeneity as well as stand productivity. Błońska et al.^4^ confirmed the positive effect of mixed-species stands on an acidification of surface soil horizons. Mixed stands compared to monoculture stands have a greater above-ground nutrient content, indicating an increase in the proportion of resources accumulated from the site and sequestration of carbon. One of the methods of diversifying the species composition of stands is the introduction of admixtures of trees and shrubs. The chemical composition of the shrub litter as well as exudations of their roots seems to influence the degree of soil enrichment and shrubs are of vital importance for the accumulation of nutrients and maintenance of soil fertility^5^. The influence of the shrubs on the soil properties differs with species because of variations in morphology, mineral absorption, composition of rooting exudations, unique microbial communities (rhizospheric, epiphytic and endophytic microorganisms), litter nutrient concentrations and mineralization, nitrogen fixation and stand ages^6–8^. Shrub influences microbial biomass and alters community composition^9^.

Polycyclic aromatic hydrocarbons (PAHs) are a group of persistent toxic pollutants that have two or more benzene rings in their structure and come mainly from the combustion of coal, gasoline, diesel and wood^10,11^. After release into the atmosphere PAHS may undergo long-range transportation and can be deposited at different distance from the source^12^. PAHs are highly hydrophobic and lipophilic and the soil has high ability to adsorb them and thus becomes an absorber for PAHs in the environment^13–15^. Since PAHs are not only toxic but also carcinogenic, their continuous accumulation in soils is a serious threat to human health and a factor limiting the growth of vegetation^16^. Thermal and moisture conditions and the intensity of insolation influence the rate of biodegradation and photolysis of PAHs in the environment^17^. The type of plant cover also affects the distribution of PAHs in soil^18^. According to Łyszczarz et al.^15^ the stand species composition plays an important role in shaping the quality and quantity of soil organic matter and soil acidification, which is reflected in the microbial activity and PAH accumulation in forest soils. Previous studies indicate that the biodegradation processes of PAHs can be influenced by the species composition of the stand^14^. Removal of PAHs is mainly regulated by the soil organic matter shaping the sorption properties^19^ and by microorganisms that promote plant growth (PGPR)^8^. The addition of biochar has positive influence on the activity of soil microorganisms, which results in more intensive removal of pollutants^20^.

Microbial degradation is considered to be one of the main methods of removing PAH from soil^21^. Understanding the mechanisms involved in microbial degradation of pollutants is the key in the bioremediation processes of contaminated areas^22^. The degradation process of PAHs can be accelerated through mycoremediation^23^. Selected species of fungi have the ability to decompose pollutants by producing lignolytic enzymes, thus can use PAHs as substrates, due to their chemical similarity to lignin^24^. Bacteria can effectively mineralize PAHs with ≤ 4 ring, while fungi can non-specifically break down PAHs of higher molecular weight^25,26^. The enzyme catechol 1,2-dioxygenase performs the first stage of aromatic ring decomposition and is associated with a number of bacteria, especially actinomycetes^27^.

The main goal of our research was to determine the impact of two shrubs species (alder buckthorn *Frangula alnus* and European hazelnut *Coryllus avellana*) in the pine forest stands on the amount of accumulated PAHs in soil. So far, no studies have been carried out on the role of shrubs in shaping the properties of forest soils, which is important for the accumulation of PAHs. Our research complements the knowledge on the role of shrubs in shaping the amount and diversity of microorganisms that are important in the degradation processes of PAHs. In our experiment, we tested the following research hypotheses: (1) shrubs, especially European hazelnut have favourable influence on the basic chemical properties of soils, which results in the biochemical activity of soils, expressed by soil enzymatic activity; (2) regardless of the species, shrubs have a beneficial effect on the activity and number of microorganisms; (3) the breeding of shrubs in pine monocultures leads to the intensification of the degradation process of PAHs as a result of stimulating the soil biological activity including microorganisms.

## Materials and methods

### Study site and experimental design

The research was carried out in the Rybnik Forest District in southern Poland (50° 05′ 55″ N; 18° 32′ 42″ E). The average annual temperature in this area is 8.4 °C, and the average annual rainfall is 705 mm. The field sites were located in an area where the soils derived from glacial sand; they are dominated by Brunic Arenosols. The Rybnik Forest District is located in one of the areas in Europe most exposed to industrial emissions. The average annual concentrations of benzo(a)pyrene (5–12 ng^/^m^3^) for the Rybnik Forest District for the years 2007–2016 exceeded the admissible standards according to Directive 2004/107/EC^28^.

Pine stands with admixture of shrubs (alder buckthorn (*Frangula alnus*) and European hazelnut (*Coryllus avellana*)) growing in the same soil conditions were selected for the study. Pine monocultures without admixtures of other species were the control variant. The research involved stands of similar age with the same tree canopy density. The age of the stands was 80 years. Each of the three variants was represented by 10 study plots. In total, 30 study areas were included in the research. Samples for analyses were collected from the organic horizon (O horizon from a depth of 0–7 cm) and humus mineral soil horizon (A horizon from a depth of 7–15 cm). Composite soil samples, consisting of 3 subsamples from different points, were collected from each horizon. The samples from each horizon were placed in a plastic container, mixed, and divided into two subsamples on which (1) soil properties and (2) metagenomic and biochemical analyses were determined. The container and tools for taking samples from profile were washed with water and ethanol (> 99% w/w) each time before the next sample was taken. All soil samples for laboratory testing were collected in June 2022.

### Laboratory analysis

The basic properties of the soil samples were determined. Using the potentiometric method, the pH of the samples was analysed in H_2_O and KCl. The organic carbon (C) and total nitrogen (N) contents were measured with an elemental analyser (LECO CNS TrueMac Analyzer; Leco, St. Joseph, MI, USA).

The PAHs were determined in 10 g of each soil sample, extracted using 70 ml (ratio of soil to extractant 1:7) of propan-2-ol for 70 min. The samples were centrifuged (4500 rpm, 5 min) and the supernatant collected. The supernatants were extracted to the solid phase (5 ml/min) using solid-phase extraction (CHROMABOND® CN/SiOH). The residue was dissolved in acetonitrile and analysed using high-pressure liquid chromatography (HPLC) with a DionexUltiMate 3000 HPLC system, equipped with a fluorescence detector and a DionexUltiMate 3000 Column Compartment C18 5 μm with a 4.6 × 100-mm HPLC column. The mobile phases were water (A) and acetonitrile (B) at a flow rate of 1 ml/min. Based on the standard PAH Calibration Mix (CRM 47940) at a concentration of 10 μg/ml, calibration solutions at different concentrations (i.e. 0.1, 0.2, 0.5, 1 and 2 μg/ml) were prepared. Each prepared solution was placed into the chromatography column, the chromatograms obtained were used to produce a calibration curve. The soil samples were then analysed in triplicate^15,29^. After every ninth analysis, a control sample (a calibration solution with a concentration of 0.1 μg/ml) was injected. Acenapthene (Ace), fluoren (Flu), phenanthrene (Phe), antracen (Ant), fluoranthene (Flt), pyrene (Pyr), benzo(a)anthracene (BaA), chrysene (Chr), benzo(k)fluoranthene (BkF),benzo(b)fluoranthene (BbF), benzo(a)pyrene (BaP), dibenzo(ah)anthracene (DBahA) indeno(1,2,3-c,d)pyren (IcdP), and bezo(g,h,i)perylene (BghiP) were determined.

The enzymatic activity was determined in fresh soil samples of natural moisture, which were passed through a sieve with a diameter of 2 mm. Soil samples for the determination of enzymatic activity were stored at 4 °C. The activity of six extracellular enzyme (β-glucosidase (BG), β-d-cellobiosidase (CB), β-xylosidase (XYL), *N*-acetyl-β-d-glucosaminidase (NAG), phosphatase (PH) and arylsulphatase (SP)) involved in the cycle of C, N and P was determined^30–32^.

### Fungal and bacterial DNA library preparation from the soil

DNA was isolated from the soil collected from the organic horizon (O, n = 3) and additionally from one sample from humus mineral soil horizon (A). DNA was isolated from 1 g of soil according to protocol of Genomic Mini AX Bacteria + (A&A Biotechnology, Poland). Mechanical lysis was carried out with zirconia balls in FastPrep-24 homogenizer. Additionally, lyticase (A&A Biotechnology, Poland) was used in enzymatic lysis.

Fungal DNA libraries were prepared for ITS1 rDNA region amplified with ITS1F^33^^`^ and ITS2 primers according to protocol “16S Metagenomic Library Preparation” (Illumina). Bacterial DNA libraries were prepared for V3-V4 16S rDNA region amplified with 341F oraz 785R primers^34^.

PCR was carried out in a reaction mixture that contained 15 ng of genomic DNA using Q5 Hot Start High-Fidelity 2× Master Mix (New England Biolabs, USA). Indexing PCR was prepared using the Nextera XT index kit (Illumina). After indexing, the samples were purified with AMPure XP beads, verified with a Bioanalyzer (Agilent Technologies, US) and qPCR. DNA libraries were sequenced on the Illumina MiSeq platform (2 × 300 bp paired end) by Genomed (Poland). Sequencing depth was 50,000 reads per sample.

### Processing of NGS data

NGS data for fungi were processed using QIIME^35^. Samples were demultiplexed and fastq files were generated with MiSeq Reporter v 2.6 (Illumina). Adapter sequences and low-quality sequences (below Q20) were removed with cutadapt^36^. Paired sequences were joined with seqprep algorithm. USEARCH was used for chimera removal^37^. Fungal reads were clustered using uclust algorithm^37^ and checked against ITS sequences UNITE v8.2 (Unite Community 2018) with the BLAST algorithm^38^. Operational taxonomic units (OTUs) were filtered for very low abundance and only OTUs with a relative abundance of at least 0.01% and a frequency of at least 3 samples were used in further analysis. The observed OTUs were used to estimate alpha diversity. Bray–Curtis and Canberra distances were calculated for beta diversity. Principal coordinates analysis was performed on the log2 transformations of OTU tables followed the formula log2(relative abundance + 1) using Bray–Curtis distances in the PAST software^39^. The R packages (https://cran.r-project.org) were applied for the analysis of microbial diversity and visualization of the results. Heatmaps were drawn using the R package superheat. The hierarchical clustering was generated based on Canberra distance method and average linkage method.

NGS data for bacteria were processed using QIIME. Samples were demultiplexed and fastq files were generated with MiSeq Reporter v 2.6 (Illumina). Adapter sequences and low-quality sequences were removed with cutadapt^36^. Paired sequences were joined and clustered with the DADA2 algorithm^40^. DADA2 was used for chimera removal. Clustered reads were checked against 16S sequences from Silva 138 database^41^. Bacterial amplicon sequence variants (ASVs) were processed and analysed as above described for fungal OTUs.

### Statistical analysis

The Pearson correlation coefficients for the soil characteristics were calculated. Principal component analysis (PCA) was used to evaluate the relationships between the soil properties and PAH content. The Shapiro–Wilk test was used to check the normal distribution. U Mann–Whitney test was used to assess the differences of properties between soil horizons (O and A horizon) while Kruskal–Wallis test was used to assess the differences between species. A general linear model (GLM) allowed to assess the importance of species and the soil depth in shaping the PAH content. Statistica 12 software (StatSoft 2012) was used for the analyzes. Differences with P < 0.05 were considered statistically significant.

## Results

### Basic properties of the soil

The influence of the studied shrub species can be seen in the basic properties of soils. Significantly lower nitrogen content was recorded in the O-horizons of soils from the alder buckthorn compare to the soil pine and in the A-horizons of pine monocultures compare to soil with shrubs (Table 1). The soils of the pine monocultures were characterized by a significantly higher carbon content in the organic horizons compare to AB and a significantly lower carbon content in the A-horizons. Significantly higher pH in H_2_O was recorded for the organic horizons of soil with the admixture of European hazelnut (Table 1). The basic properties of the studied soils changed with the depth. At deeper horizons (A), lower N and C content and a decrease in pH KCl were noted.

**Table 1:** Basic properties and PAH content (μg/kg) in soil under influence of shrubs.

Species	Horizon	N (%)	C (%)	C/N	pH H_2_O	pHKCl	sum PAH
AB	O	0.95 ± 0.22^bX^	20.76 ± 4.11^bX^	21.76 ± 1.17^abX^	3.97 ± 0.14^bX^	3.38 ± 0.14^aY^	161.49 ± 126.62^bX^
	A	0.29 ± 0.04^aY^	7.14 ± 1.18^aY^	23.89 ± 0.50^bX^	3.79 ± 0.09^aY^	3.51 ± 0.06^aX^	105.94 ± 34.04^bX^
EH	O	1.03 ± 0.19^abX^	21.42 ± 3.95^abX^	20.74 ± 0.52^bY^	4.22 ± 0.10^aX^	3.46 ± 0.13^aX^	65.89 ± 25.51^bX^
	A	0.44 ± 0.08^aY^	10.28 ± 2.04^aY^	23.33 ± 0.82^bX^	3.78 ± 0.04^aY^	3.35 ± 0.07^bX^	24.41 ± 12.17^cY^
SP	O	1.24 ± 0.03^aX^	28.48 ± 0.49^aX^	22.85 ± 0.31^aY^	3.98 ± 0.05^bX^	3.35 ± 0.03^aX^	635.99 ± 270.26^aX^
	A	0.16 ± 0.05^bY^	4.24 ± 1.46^bY^	26.40 ± 0.91^aX^	3.66 ± 0.05^aY^	3.19 ± 0.05^cY^	655.86 ± 362.01^aX^

mean ± SD; AB, alder buckthorn; EH, European hazelnut; SP, Scots pine; small letters in the upper index (a,b,c) mean significant differences between different species; big letters in the upper index (X,Y,Z) mean significant differences between different horizons.

### PAH content

**Figure 1:**
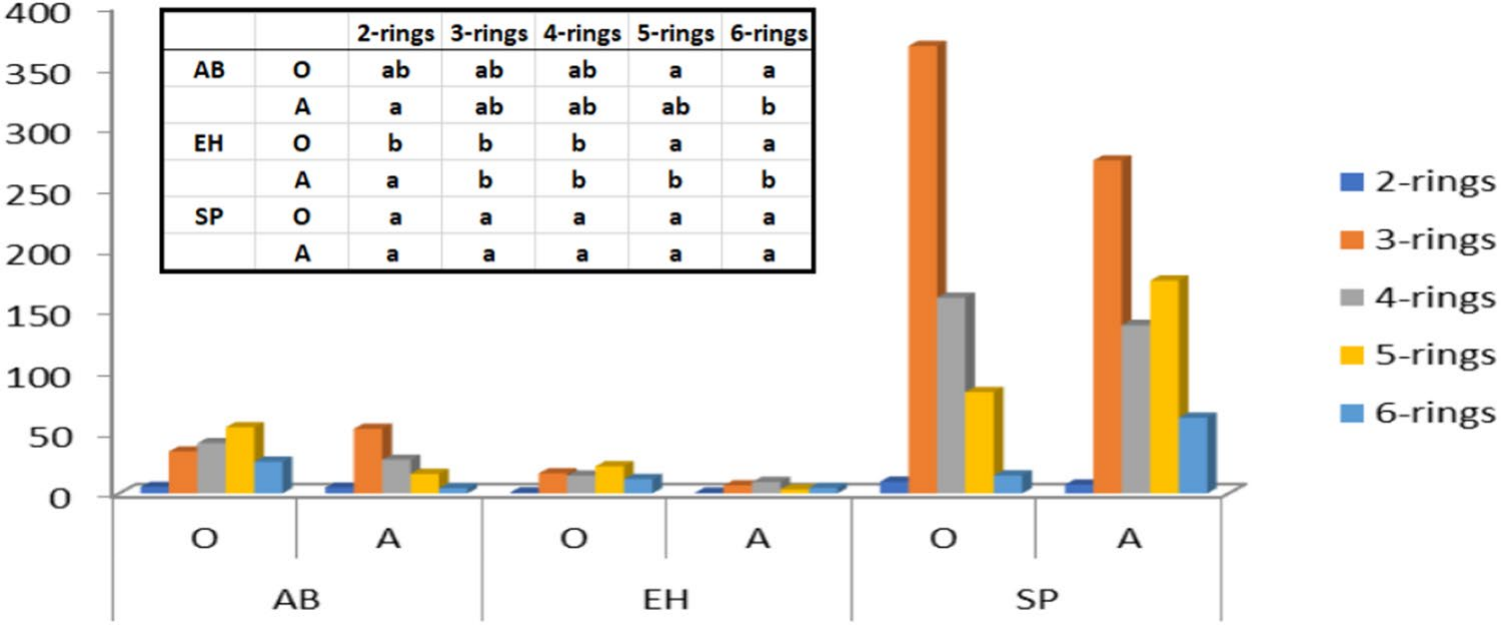
PAH content (μg/kg) taking into account the number of rings in the different soil horizons (O and A) depending on the benzene rings; AB—alder buckthorn, EH—European hazelnut, SP—Scots pine; small letters in the upper index (a, b) mean significant differences between different species.

The studied soils differed significantly in the content of PAH (Table 1). Significantly lower PAH content was found in soils with shrubs, regardless of the soil horizon. The statistical lowest PAH accumulation in A horizon was found in soils with stands with an admixture of European hazelnut. In the O horizon of the European hazelnut stands, the mean PAH content was 65.89 μg/kg, and in the A horizons it was 24.41 μg/kg. In the soils of pine monocultures, the PAH content was 10 times higher (Table 1). In the case of pine stands with an admixture of EH, the PAH content decreased significantly with soil depth. A lower PAH content was noted in the humus mineral horizons (A) compared to the organic horizons (O). In the case of pine monoculture soils without admixtures of shrubs, the PAH content did not differ within the studied horizons. 3-, 4- and 5-ring hydrocarbons were predominant in the investigated soils independently of the analyzed horizon of soil and species (Fig. 1). In the soils of the investigated tree stands no 2-ring PAHs could be found and the contribution of 6-ring hydrocarbons was minor (Fig. 1).

### Enzymatic activity

**Figure 2:**
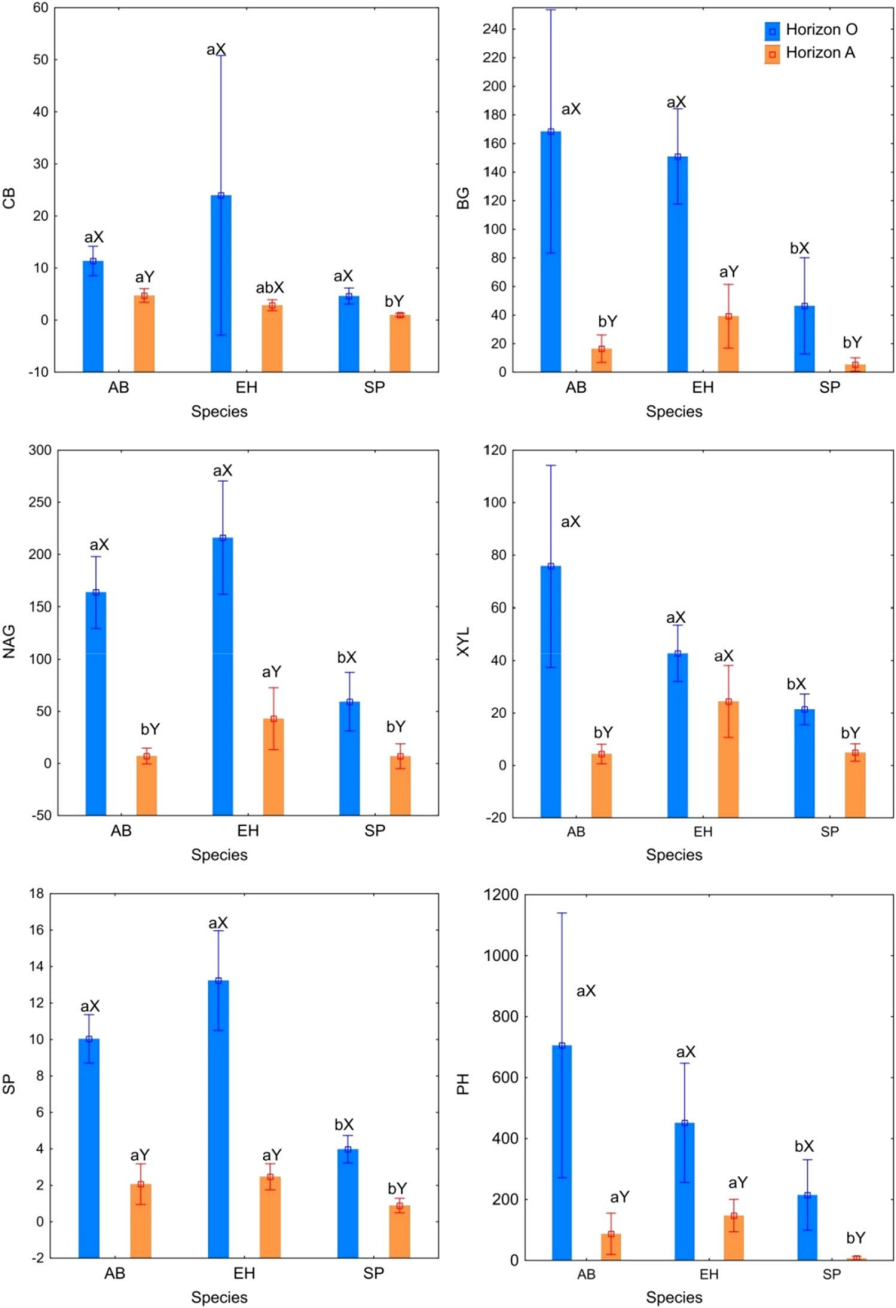
Enzyme activity in soil under influence different species; mean and SD; CB—β-d-cellobiosidase, BG—β-glucosidase, NAG—*N*-acetyl-β-Glucosaminidase, XYL—β-Xylosidase, SP—sulphatase, PH—phosphatase; AB—alder buckthorn, EH—European hazelnut, SP—Scots pine; small letters in the upper index (a, b, c) mean significant differences between different species; big letters in the upper index (X, Y) mean significant differences between different horizons.

**Figure 3:**
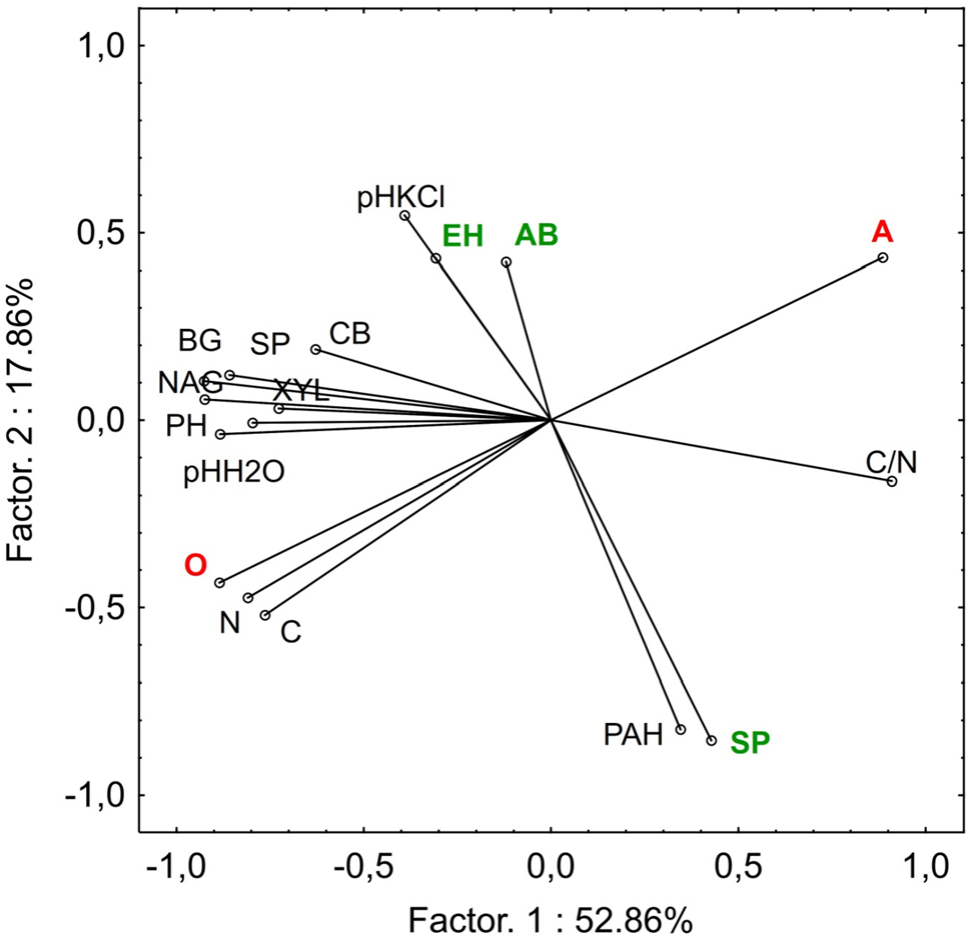
The projection of variables on a plane of the first and second PCA factor; CB—β-d-cellobiosidase, BG—β-glucosidase, NAG—*N*-acetyl-β-glucosaminidase, XYL—β-Xylosidase, SP—sulphatase, PH—phosphatase; AB—alder buckthorn, EH—European hazelnut, SP—Scots pine; O—organic horizon, A—humus mineral horizon.

The activity of some enzymes in the studied soils was diversified. Along with the depth, a significant decrease in the activity of the tested enzymes was noted, often several times (Fig. 2). In soils with shrubs, the activity of most of the tested enzymes, except CB, was found to be significantly higher. The activity of BG, NAG, XYL, SP, PH in the soils of pine monocultures without admixture of shrubs was 3–4 times lower compared to the soils of stands with shrubs (Fig. 2). The activity of most of the tested enzymes (BG, NAG, XYL, PH and SP) positively correlated with the content of N, C and pH in H_2_O (Table 2). All tested enzymes significantly negatively correlated with the C/N ratio. In the case of BG, NAG and SP activity, a negative correlation was found with the PAH content. GLM analysis confirmed the significance of species and soil depth in the shaping of enzymes activity (Table 3). The GLM analysis performed showed the combined effect of species and soil depth in shaping the activity of most of the studied enzymes, apart from CB activity. In addition, significance of species in the formation of PAHs amount was revealed (Table 3). PCA analysis confirmed a clear relationship between the PAH content, enzymatic activity and basic properties of the studied soils (Fig. 3). Two basic factors had a significant impact on the property variance (70.7%). PCA indicated a strong relationship between the soils of pine monoculture stands without the admixture of shrubs with high accumulation of PAHs. The PCA clearly separated the soils of the stands with the admixture of shrubs and the soil of pine monocultures. The conducted PCA analysis confirmed the distinctiveness in terms of the properties of the genetic levels of the studied soils (Fig. 3).

**Table 2:** Pearson correlation coefficients between enzyme activity and physicochemical properties, PAHs content in the soils under influence different species.

	N	C	C/N	pH H_2_O	pHKCl	sum PAH
CB	0.30	0.26	− 0.55*	0.53*	0.26	− 0.29
BG	0.56*	0.50*	− 0.74*	0.66*	0.30	− 0.39*
NAG	0.64*	0.58*	− 0.78*	0.78*	0.19	− 0.41*
XYL	0.48*	0.47*	− 0.53*	0.44*	0.08	− 0.25
SP	0.61*	0.55*	− 0.78*	0.80*	0.33	− 0.42*
PH	0.64*	0.59*	− 0.70*	0.65*	0.22	− 0.22
sum PAH	0.04	0.08	0.46*	-0.28	-0.57*	1.00

CB, β-d-cellobiosidase; BG, β-Glucosidase; NAG, *N*-acetyl-β-glucosaminidase; XYL, β-xylosidase; SP, sulphatase; PH, phosphatase.

*Significance effect (P < 0.05).

**Table 3:** Summary of GLM analysis of the effect of the shrubs species and soil horizon on the PAH content and enzyme activity.

	CB		BG		NAG		XYL		SP		PH		sum PAH	
	F	p-value	F	p-value	F	p-value	F	p-value	F	p-value	F	p-value	F	p-value
Species (S)	3.54	**0.0447**	14.08	**0.0001**	35.98	**0.0001**	9.91	**0.0007**	59.94	**0.0001**	7.85	**0.0001**	25.77	**0.0001**
Horizon (H)	10.36	**0.0036**	70.88	**0.0001**	187.74	**0.0001**	47.27	**0.0000**	32.58	**0.0001**	39.69	**0.0001**	0.12	0.7303
S*H	2.74	0.0841	7.22	**0.0001**	16.52	**0.0003**	12.25	**0.0002**	29.67	**0.0001**	4.30	**0.0252**	0.09	0.9065

CB, β-d-cellobiosidase; BG, β-glucosidase; NAG, *N*-acetyl-β-glucosaminidase; XYL, β-xylosidase; SP, sulphatase; PH, phosphatase.

Significant values are in [bold].

### Fungal diversity

**Figure 4:**
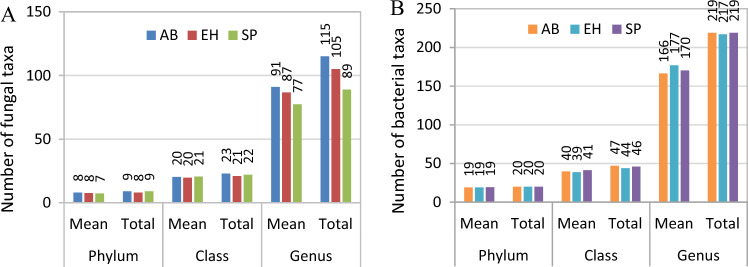
Mean and total number of fungal (**A**) and bacterial (**B**) taxa identified in soil organic horizon under influence different species; AB—alder buckthorn, EH—European hazelnut, SP—Scots pine.

A total of 634,528 reads (with acceptable quality) of fungal ITS1 rDNA from soil samples were obtained. The average number of reads per sample was 57,847. We identified comparable total number of fungal phyla and classes in soil samples from O horizon under influence of shrubs alder buckthorn and European hazelnut and Scots pine monoculture. There were respectively 8, 8 and 7 phyla and 23, 21 and 22 classes. The total number of fungal genera under influence of alder buckthorn and European hazelnut was 29% and 10% higher than in the pine monoculture (respectively, 115 and 105 versus 89 genera; Fig. 4A). Sixty seven of total 127 genera were common for all sites. The number of unique genera was very low and reached 3 for AB, 4 for EH and 5 for SP site. The number of taxa common for AB and EH was 31, for AB and SP was 8 and for EH and SP was 3. The mean number of fungal phyla ranged from 7 to 8, classes—from 21 to 23 and genera—from 77 to 91 and did not differ between study sites (Fig. 4A).

**Figure 5:**
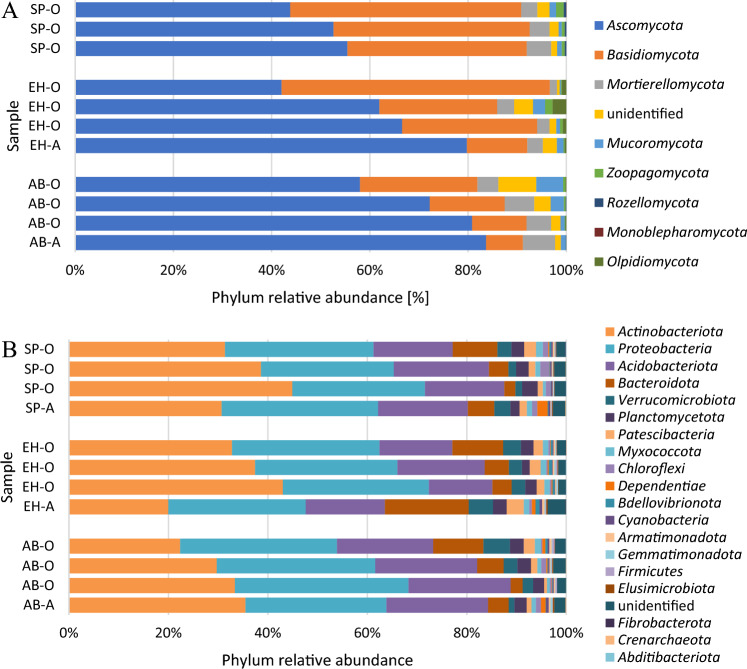
Relative abundance of fungal (**A**) and bacterial (**B**) phyla in soil (O—organic horizon, A—humus mineral horizon) under influence different species; AB—alder buckthorn, EH—European hazelnut, SP—Scots pine.

The fungal community of the soil (O horizon) was dominated by *Ascomycota* phylum (alder buckthorn—mean relative abundance 70%, European hazelnut—57%, Scots pine—51%), with *Basidiomycota* being the second most abundant phylum (respectively, 17%, 35% and 41%) (Fig. 5A). Relative abundances of other phyla were significantly lower: *Mortierellomycota* (respectively, 4%, 2% and 2%)*,* unidentified phylum (respectively, 4%, 2% and 2%), *Mucoromycota* (respectively, 3%, 1% and 1%), *Zoopagomycota, Rozellomycota, Monoblepharomycota* and *Olpidiomycota* (accounted for less than 1%).

**Figure 6:**
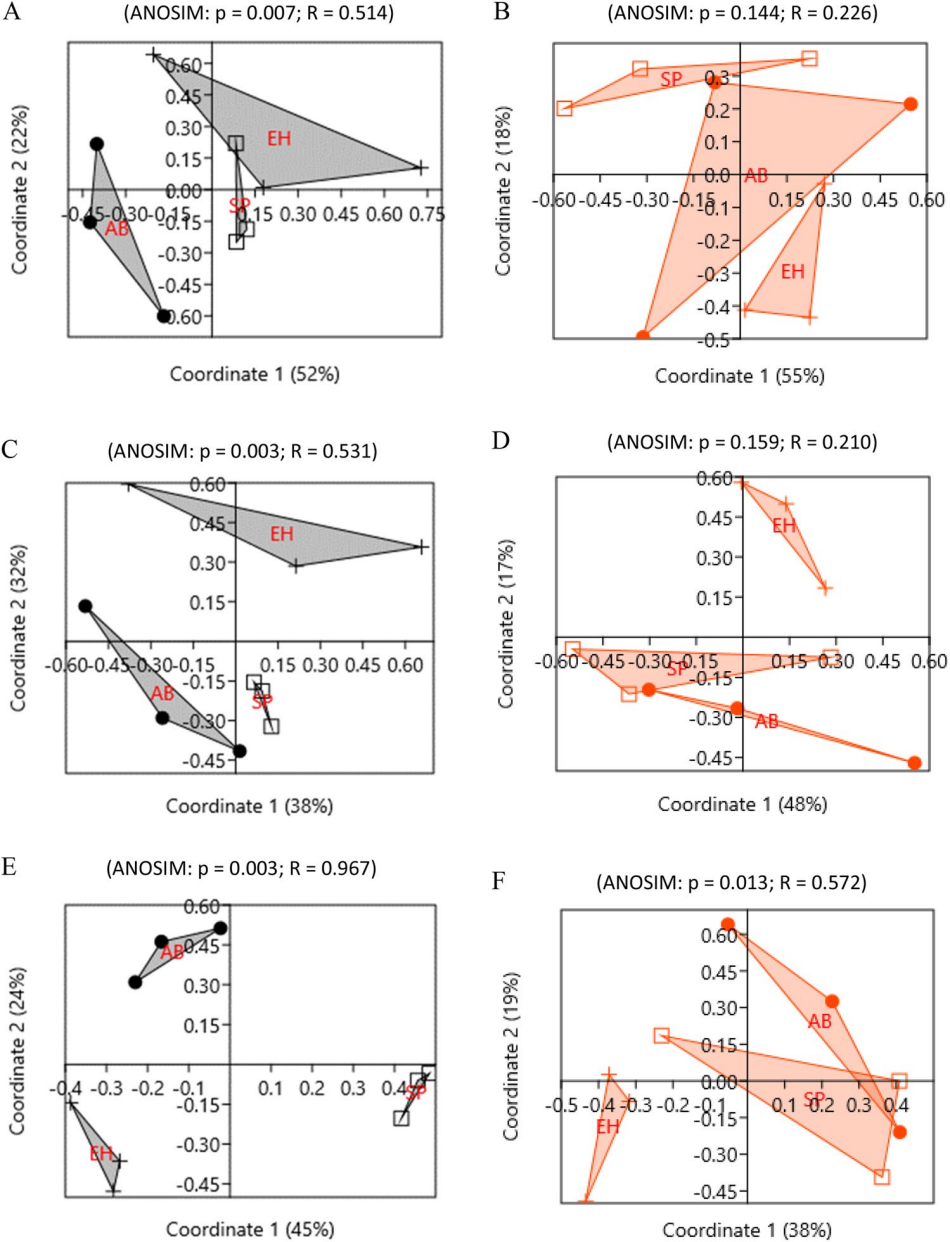
Principal coordinates analysis (PCoA) based on Bray–Curtis distances between samples of soil organic horizon under influence different plant species; AB—alder buckthorn, EH—European hazelnut, SP—Scots pine calculated for phylum (**A**—fungi; B—bacteria) and class (**C**—fungi; **D**—bacteria) and genus (**E**—fungi; **F**—bacteria) level (log_2_(RA + 1)).

Principal coordinate analysis (PCoA) based on Bray–Curtis distances showed that the compositions of the fungal community in the soil from O horizon under influence of alder buckthorn and European hazelnut admixture and Scots pine monoculture varied (Fig. 6). ANOSIM analysis revealed that the differences were statistically significant at the genus level (*p* = 0.003; Fig. 6E), at the class level (*p* = 0.003; Fig. 6C) and even at the phylum level (*p* = 0.007; Fig. 6A).

**Figure 7:**
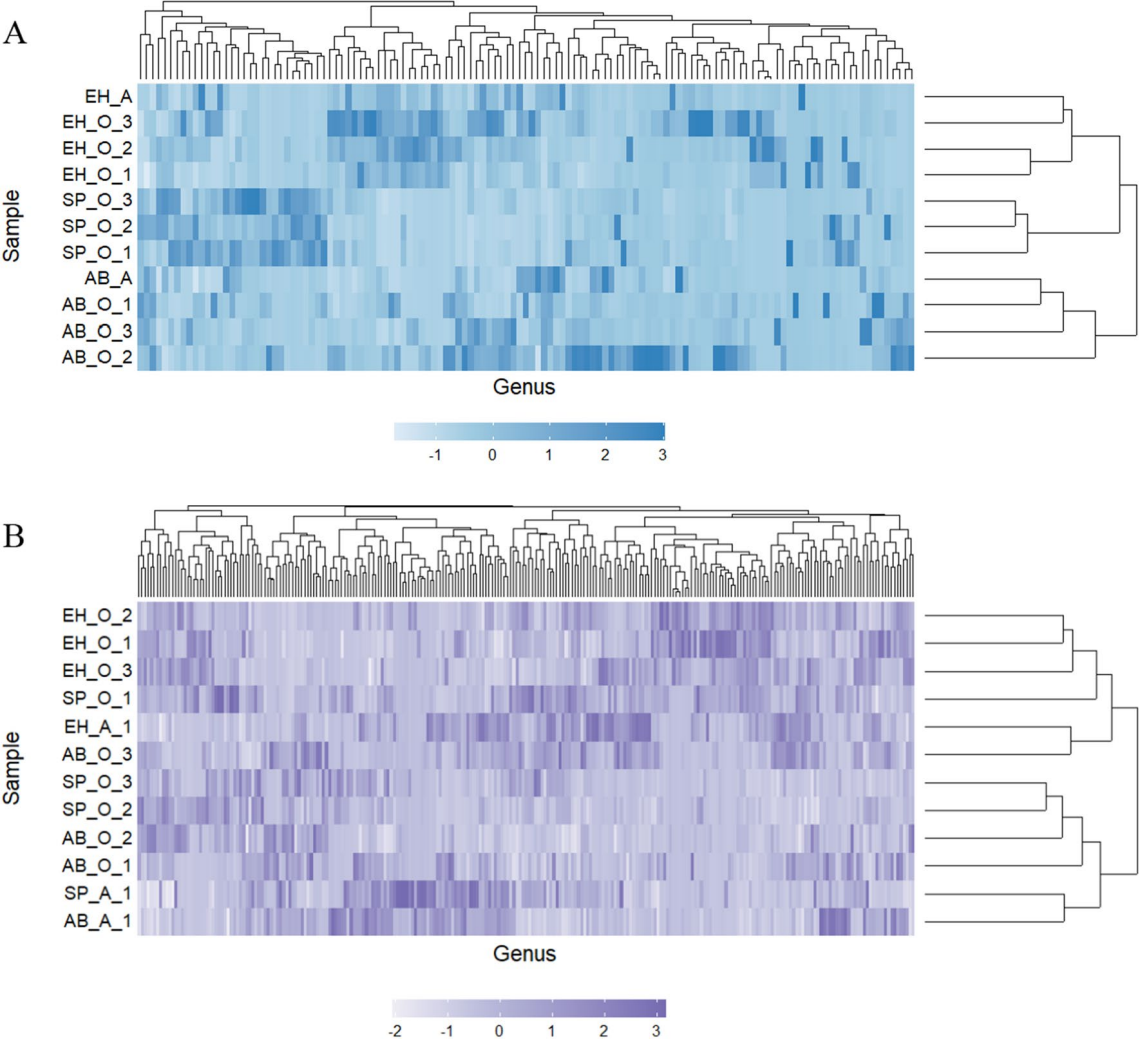
Heatmap illustrating the relative abundance of the fungal (**A**) and bacterial (**B**) genera in all samples used in this study with dendrograms calculated based on Canberra distances between samples; AB—alder buckthorn, EH—European hazelnut, SP—Scots pine, O—organic horizon, A—humus mineral horizon.

Canberra distance-based clustering clearly showed that samples belonging to the soil from Pine admixtured with shrubs of alder buckthorn and European hazelnut and Scots pine monoculture were clustered separately to three distinct clusters corresponding to experimental variants (Fig. 7A). Moreover, single samples from A horizon investigated for AB and EH sites were clustered with O horizon samples of corresponding sites.

**Figure 8:**
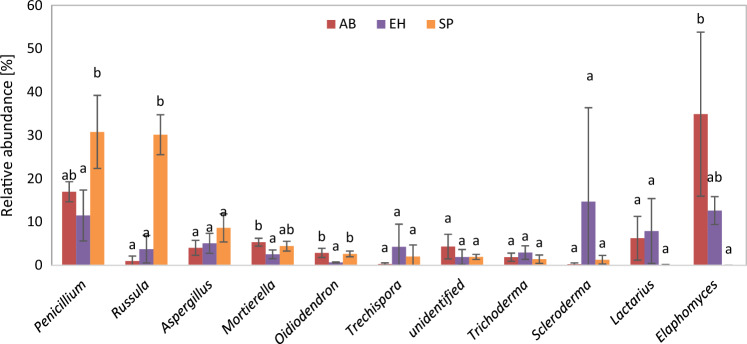
Relative abundance of the most dominant fungal genera in soil (organic horizon) under influence different species; AB—alder buckthorn, EH—European hazelnut, SP—Scots pine. The different letters mean differences between study site for particular genera of fungi.

We compared relative abundances of the most dominant fungal genera comprising *Penicillium, Russula, Aspergillus, Mortierella, Oidiodendron, Trechispora, Trichoderma, Scleroderma, Lactarius, Elaphomyces* and unidentified genus between study sites (Fig. 8). Control site was dominated by *Penicillium* genus (31%)*.* Relative abundance of *Penicillium* in control site (SP) was significantly higher than in AB (11%) and did not differ with EH sites (17%). *Russula* was the second most dominant genus in SP monoculture site. Its relative abundance equalled 30% and was significantly higher than in sites with shrubs: alder buckthorn, (1%) and European hazelnut (4%). Relative abundances of *Mortierella*, *Oidiodendron* and *Elaphomyces* was significantly higher in AB site (respectively, 5%, 3% and 35%) in comparison to EH site (respectively, 3%, 1% and 0%). No significant differences were observed in relative abundances of other genera between study sites.

### Bacterial diversity

A total of 550,385 reads (with acceptable quality) of bacterial 16S rDNA from soil samples were obtained. The average number of reads per sample was 45,865. The total number of bacterial taxa, similarly to fungal taxa, was comparable between study sites. The total number of phyla was 20 in each site, the total number of classes ranged from 44 (European hazelnut) to 47 (alder buckthorn) and the total number of genera ranged from 217 (European hazelnut) to 219 (alder buckthorn, Scots pine) in O soil horizon (Fig. 4B). The mean number of phyla, classes and genera equalled respectively 20, 39–41 and 166–177 (Fig. 4B).

Soil bacteria microbiome was dominated by 3 phyla: *Actinobacteriota, Proteobacteria* and *Acidobacteriota* (cumulative relative abundances of these phyla were over 80% for each site). The mean relative abundance of *Actinobacteriota* was 28% in AB site and 38% in EH and SP sites (Fig. 5B). *Proteobacteria* was accounted on 33% in AB site, 29% in EH site and 28% in SP site. *Acidobacteriota* was accounted on 20%, 15% and 17%, respectively. The relative abundances of phyla *Bacteroidota, Verrucomicrobiota, Planctomycetota, Patescibacteria* and unidentified phylum ranged from 6 to 1%. The ratio of other phyla accounted for 1 or less per cent (Fig. 5B).

Principal coordinate analysis (PCoA) based on Bray–Curtis distances showed that the composition of the bacterial community in the soil from O horizon under influence of admixture of alder buckthorn and European hazelnut and Scots pine monoculture did not differ at phylum and class level (Fig. 6B and D). ANOSIM analysis revealed that the differences were statistically significant at the genus level (*p* = 0.013 Fig. 6F). Bacterial community in soil under European hazelnut differed from bacterial community of control site (Scots pine) more pronounced than bacterial community in soil under alder buckthorn (Fig. 6F).

Heatmap with dendrograms showed that samples belonging to soil under influence of shrubs, alder buckthorn and European hazelnut and Scots pine monoculture were not clustered separately (Fig. 7B).

## Discussion

The conducted research confirmed the correctness of the formulated hypotheses. The species composition of stands with admixture of shrubs were important in shaping soil properties, especially PAH accumulation. Soils with an admixture of shrubs, regardless of the species, were characterized by the lowest accumulation of PAHs in soils. The soils of pine stands were characterized by a ten-fold higher accumulation of PAHs compared to the soils of forest stands with an admixture of European hazelnut. Earlier studies indicate the influence of stand species composition on PAH accumulation in forest soils, which is related to the different influence of deciduous and coniferous species on basic soil properties such as pH and quality of soil organic matter^14,15,42^. The organic horizon of pine stands with an admixture of European hazelnut were characterized by a significantly higher pH and the lowest C/N ratio. Aromatic carbon structures in soil organic carbon (SOC) plays an important role in the adsorption of PAHs, and acidification of the soil environment affects the activity of microorganisms indirectly influencing the accumulation of PAHs^43,44^. According to Hui et al.^43^ factors such as soil acidification, change in the content of TOC, TN and P and the reduced electron transfer capacity of DOM may reduce the activity of soil microorganisms and enzymes, and thus reduce degradation of PAHs. Sorption by soil organic matter is considered to be the most important process influencing the bioavailability of hydrophobic organic compounds in soil^45^. In our research, we noted a different amount and quality of soil organic matter expressed by the C/N ratio. The soils of the stands with an admixture of shrubs, especially with European hazelnut, were characterized by a better decomposed organic matter, which was reflected by a narrower C/N ratio. The soils of the pine stands without the admixture of shrubs were characterized by more acidified and less decomposed organic matter accompanied by lower enzymatic activity, which resulted in a higher accumulation of PAHs. Different plant communities with different microbial metabolism differed in the activity of soil enzymes, and they can change the soil environment in different ways^46^. Vegetation influences the microbial diversity and enzymatic activity by influencing soil C and stocks of nutrients^47^. Tree species may differ in the chemical and physical properties of the litter, the depth of decomposition and root rotation, and their effects on the microclimate and the ability to redistribute nutrients^48^. The results of these authors indicate that species with high quality bedding have fewer nutrient limitations, which results in faster degradation as a result of an increase in microbial activity. Ghosal et al.^49^ described a large number of microorganisms that degrade PAHs in combination with the appropriate conditions necessary for degradation, i.e. pH, temperature and nutrient availability.

Admixture of shrubs in pine stands had beneficial effect on the number of fungal taxa identified in the soil and changed the structure of their communities. The number of genera in pine stands admixtured with European hazelnut was 29% higher than in stand without shrubs. In stand admixture with alder buckthorn it was 10%. In the soil of pine stands with the participation of alder buckthorn and hazelnut, a greater share of certain groups of microorganisms is visible. The proportion of Ascomycota was almost twice or more as high as that of Basidiomycota fungi in stands admixtured with shrubs. In pine stands without shrubs proportion of these phyla was comparable. The dominance of the Ascomycota group was also found in the study of the structure of fungal populations in the root zone of various species of hazelnut^50^. It seems that high rate of this phylum is vulnerable from the point of view the ability of fungi to decompose of PAHs. According to the research by Aranda^25^, fungi that belong to Ascomycota and Mortierellomycotina are capable of decomposing PAHs, which may explain the lower concentration of these pollutants in the soils of forest stands with the participation of shrubs. It is also possible not to exclude the positive impact of shrubs on the overall biological activity and the increase in the rate of decomposition of organic matter, and thus the acceleration of the degradation rate of harmful organic pollutants associated with organic matter as multiple soil functions are dependent on decomposition of organic matter^51^. In the structure of bacteria present in the soil of stands with the participation of shrubs (alder buckthorn and European hazelnut), a higher number of bacteria from the Bacteroidota and Verrucomicrobiota phyla was found. Bacteria degrade PAH compounds by an assimilative process where they gain carbon and energy for the growth, which typically leads to mineralization of the compound^52^. Bacteria generally use intracellular dioxygenase enzymes for the degradation of PAHs^53^. The Bacteroidota phylum is of great importance in the degradation of chitin in the soil environment^54^, while the Verrucomicrobiota group are involved in the hydrolysis of polysaccharides^55^ and significantly participate in the transformation of nitrogen compounds in the soil^56^ stimulating the development of soil nematodes^57,58^. A positive effect on the development of nematodes, as well as an increase in the rate of conversion of polysaccharides and nitrogen compounds, have a significant impact on the rate of mineralization of soil organic matter. The aforementioned bacterial clusters have shown a significantly higher number in the soil of pine stands with hazelnut, in the humus-mineral horizon, in which the roots of the examined shrubs develop intensively, which may indicate that this species has a different effect on the structure of the bacterial population, at the same time shaping the intensity of biochemical changes in soil environment. The analysis of the dominant fungal genera in soil samples taken from the organic horizons of the studied soils revealed significant differences in the composition of the dominant taxa, probably related to the type of detritus reaching the soil. Strong dominance of two genera, *Penicillium* and *Russula* (cumulative RA over 60%) in stands without shrubs have indicated on lower evenness of fungi in comparison with stands admixture with shrubs, especially European hazelnut, where the number of main players was highest. In pine stands with alder buckthorn and hazelnut, the fungi of the genera *Scleroderma, Lactarius* and *Elaphomyces* were numerous, with the first type showing a higher number in patches of stands with hazelnut, and the third in patches of stands with alder buckthorn. According to Kadri et al.^59^ the importance of fungi in PAH degradation is related to the ability of some fungi to hydroxylate a wide range of PAHs and their ecological role may be significant as these polar intermediates can be mineralized by soil bacteria or detoxified to simpler, harmless compounds. Additionally, fungi have an advantage over bacteria because mycelium can grow into the soil and spread by breaking down PAHs. Exudates secreted in the rhizosphere zone indirectly affect the biodegradation processes of PAHs in the soil environment. Earlier studies indicate that up to 21% of all assimilates formed in the process of photosynthesis are delivered with exudate to the rhizosphere zone, which affects microorganisms and their activity^60^.

## Conclusions

Our research indicates the importance of shrubs in shaping the properties of surface horizons of forest soil and, consequently on the accumulation of PAHs. Soils of stands with an admixture of shrubs, especially hazelnut, were characterized by the highest enzymatic activity with the lowest accumulation of PAHs. The accumulation of PAHs in the soils of pine stands was 10 times higher than in the soils of stands with an admixture of shrubs. The share of shrubs in pine stands increases the diversity of soil microorganisms, which are characterized by the ability to biodegrade PAHs. In areas exposed to PAH contamination, attention should be paid to the species composition of stands which should be diversified by introducing shrubs.

## Data Availability

Data will be made available on request. Contact person—Ewa Błońska.
